# Associations between Intimate Partner Violence and Health among Men Who Have Sex with Men: A Systematic Review and Meta-Analysis

**DOI:** 10.1371/journal.pmed.1001609

**Published:** 2014-03-04

**Authors:** Ana Maria Buller, Karen M. Devries, Louise M. Howard, Loraine J. Bacchus

**Affiliations:** 1 Gender Violence and Health Centre, Department of Global Health and Development, Faculty of Public Health and Policy, London School of Hygiene & Tropical Medicine, London, United Kingdom; 2 Section of Women's Mental Health and King's Health Partners Women's Health Academic Centre, Institute of Psychiatry, King's College London, London, United Kingdom; University of California, San Francisco, United States of America

## Abstract

**Background:**

Intimate partner violence (IPV) among men who have sex with men (MSM) is a significant problem. Little is known about the association between IPV and health for MSM. We aimed to estimate the association between experience and perpetration of IPV, and various health conditions and sexual risk behaviours among MSM.

**Methods and Findings:**

We searched 13 electronic databases up to 23 October 2013 to identify research studies reporting the odds of health conditions or sexual risk behaviours for MSM experiencing or perpetrating IPV. Nineteen studies with 13,797 participants were included in the review. Random effects meta-analyses were performed to estimate pooled odds ratios (ORs). Exposure to IPV as a victim was associated with increased odds of substance use (OR = 1.88, 95% CI_OR_ 1.59–2.22, *I* ^2^ = 46.9%, 95% CI*_I_* ^2^ 0%–78%), being HIV positive (OR = 1.46, 95% CI_OR_ 1.26–1.69, *I* ^2^ = 0.0%, 95% CI*_I_* ^2^ 0%–62%), reporting depressive symptoms (OR = 1.52, 95% CI_OR_ 1.24–1.86, *I* ^2^ = 9.9%, 95% CI*_I_* ^2^ 0%–91%), and engagement in unprotected anal sex (OR = 1.72, 95% CI_OR_ 1.44–2.05, *I* ^2^ = 0.0%, 95% CI*_I_* ^2^ 0%–68%). Perpetration of IPV was associated with increased odds of substance use (OR = 1.99, 95% CI_OR_ 1.33–2.99, *I* ^2^ = 73.1%). These results should be interpreted with caution because of methodological weaknesses such as the lack of validated tools to measure IPV in this population and the diversity of recall periods and key outcomes in the identified studies.

**Conclusions:**

MSM who are victims of IPV are more likely to engage in substance use, suffer from depressive symptoms, be HIV positive, and engage in unprotected anal sex. MSM who perpetrate IPV are more likely to engage in substance use. Our results highlight the need for research into effective interventions to prevent IPV in MSM, as well as the importance of providing health care professionals with training in how to address issues of IPV among MSM and the need to raise awareness of local and national support services.

*Please see later in the article for the Editors' Summary*

## Abstract 2

Ana Maria Buller and colleagues review 19 studies and estimate the associations between the experience and perpetration of intimate partner violence and various health conditions and sexual risk behaviors among men who have sex with men.

*Please see later in the article for the Editors' Summary*

## Editors' Summary

**Background:**

Intimate partner violence (IPV, also called domestic violence) is a common and widespread problem. Globally, nearly a third of women are affected by IPV at some time in their life, but the prevalence of IPV (the proportion of the population affected by IPV) varies widely between countries. In central sub-Saharan Africa, for example, nearly two-thirds of women experience IPV during their lifetime, whereas in East Asia only one-sixth of women are affected. IPV is defined as physical, sexual, or emotional harm that is perpetrated on an individual by a current or former partner or spouse. Physical violence includes hitting, kicking, and other types of physical force; sexual violence means forcing a partner to take part in a sex act when the partner does not consent; and emotional abuse includes threatening a partner by, for example, stalking them or preventing them from seeing their family. The adverse effects of IPV for women include physical injury, depression and suicidal behaviour, and sexual and reproductive health problems such as HIV infection and unwanted pregnancies.

**Why Was This Study Done?:**

IPV affects men as well as women. Men can be subjected to IPV either by a female partner or by a male partner in the case of men who have sex with men (MSM, a term that encompasses homosexual, bisexual, and transgender men, and heterosexual men who sometimes have sex with men). Recent reviews suggest that the prevalence of IPV in same-sex couples is as high as the prevalence of IPV for women in opposite-sex relationships: reported lifetime prevalences of IPV in homosexual male relationships range between 15.4% and 51%. Little is known, however, about the adverse health effects of IPV on MSM. It is important to understand how IPV affects the health of MSM so that appropriate services and interventions can be provided to support MSM who experience IPV. In this systematic review (a study that identifies all the research on a given topic using predefined criteria) and meta-analysis (a study that combines the results of several studies using statistical methods), the researchers investigate the associations between the experience and perpetration of IPV and various health conditions and sexual risk behaviours among MSM.

**What Did the Researchers Do and Find?:**

The researchers identified 19 studies that investigated associations between IPV and various health conditions or sexual risk behaviours (for example, unprotected anal sex, a risk factor for HIV infection) among MSM. The associations were expressed as odds ratios (ORs); an OR represents the odds (chances) that an outcome will occur given a particular exposure, compared to the odds of the outcome occurring in the absence of that exposure. The researchers estimated pooled ORs from the data in the individual studies using meta-analysis. The pooled lifetime prevalence of experiencing any IPV (which was measured in six studies) was 48%. Exposure to IPV as a victim was associated with an increased risk of substance (alcohol or drug) use (OR = 1.88, data from nine studies), reporting depressive symptoms (OR = 1.52, data from three studies), being HIV positive (OR = 1.46, data from ten studies), and engagement in unprotected sex (OR = 1.72, data from eight studies). Perpetration of IPV was associated with an increased risk of substance abuse (OR = 1.99, data from six studies).

**What Do These Findings Mean?:**

These findings suggest that MSM frequently experience IPV and that exposure to IPV is associated with several adverse health conditions and sexual risk behaviours. There were insufficient data to estimate the lifetime prevalence of IPV perpetration among MSM, but these findings also reveal an association between IPV perpetration and substance use. The accuracy of these findings is limited by heterogeneity (variability) between the studies included in the meta-analyses, by the design of these studies, and by the small number of studies. Despite these and other limitations, these findings highlight the need to undertake research to identify interventions to prevent IPV among MSM and to learn more about the health effects of IPV among MSM. They highlight the importance of health care professionals being aware that IPV is a problem for MSM and of training these professionals to assess MSM for IPV. Finally, these results highlight the need to improve the availability and effectiveness of support services to which health care professionals can refer MSM experiencing or perpetrating IPV.

**Additional Information:**

Please access these websites via the online version of this summary at http://dx.doi.org/10.1371/journal.pmed.1001609.

- The World Health Organization provides detailed information on intimate partner violence
- The US Centers for Disease Control and Prevention provides information about IPV and a fact sheet on understanding IPV that includes links to further resources
- The UK National Health Service Choices website has a webpage about domestic violence, which includes descriptions of personal experiences
- The US National Domestic Violence Hotline provides confidential help and support to people experiencing IPV, including MSM; its website includes personal stories of IPV
- The US Gay Men's Domestic Violence Project/GLBTQ Domestic Violence Project provides support and services to MSM experiencing IPV; its website includes some personal stories
- The UK not-for-profit organization Respect runs two advice lines: the Men's Advice Line provides advice and support for men experiencing domestic violence and abuse and the Respect Phoneline provides advice for domestic violence perpetrators and for professionals who would like further information about services for those using violence/abuse in their intimate partner relationships
- The UK not-for-profit organization ManKind Initiative also provides support for male victims of IPV
- The UK not-for-profit organization Broken Rainbow UK provides help and support for lesbians and MSM experiencing IPV
- MedlinePlus provides links to other resources about domestic violence (in English and Spanish)
- The UK charity Galop gives advice and support to people who have experienced biphobia, homophobia, transphobia, sexual violence, or domestic abuse

## Introduction

Violence by an intimate partner affects nearly one in three women globally, ranging from 16.3% in East Asia to 65.6% in central sub-Saharan Africa [1]–[3]. The associated adverse health consequences for female victims include depression, anxiety, post-traumatic stress disorder, eating disorders [4],[5], and sexual and reproductive health problems [6],[7]. However, intimate partner violence (IPV) is not exclusive to opposite-sex relationships, and there is a growing body of research highlighting the prevalence of IPV in same-sex relationships [8]–[14]. Recent reviews suggest that the prevalence in same-sex couples, in particular male–male couples, is as high as or higher than it is for women in opposite-sex relationships [15]–[18]. The reported lifetime experience of IPV in gay male relationships lies between 15.4% and 51% [9],[11],[15],[18], depending on the population studied [11],[17], the definition of “partner” or “relationship” [11], and the type of measures used [19]. Most reviews addressing of IPV in the LGBT (lesbian, gay, bisexual, and transgender) population focus on the prevalence of IPV, with limited research on the health associations of IPV [20]. The two existing reviews that explored this association [11],[17] used a narrative approach, which summarises and explains results in words rather than pooling quantitative results, and concluded that further research was needed to understand the range of health conditions associated with IPV among MSM.

A UK study on safety planning and advocacy services for men affected by IPV, found that gay and heterosexual men need different services and approaches to accessing support [21]. Insight into IPV health associations for this population will both inform the identification of opportunities for intervention, and guide the design of tailored interventions. To our knowledge this systematic review and meta-analysis is the first to quantitatively synthesise evidence on the association between exposure to IPV as a victim and a perpetrator with health conditions and sexual risk behaviours among men who have sex with men (MSM). We aimed to assess whether MSM who experience or perpetrate IPV have increased odds of common mental health disorder symptoms, eating disorder symptoms, substance misuse, sexually transmitted infections (STIs), HIV positive status, or sexual risk-taking behaviours compared with MSM who are not experiencing or perpetrating IPV.

## Methods

The review followed PRISMA [22] and MOOSE [23] guidelines (see Table S1 for the PRISMA checklist). Thirteen bibliographic databases (MEDLINE, EMBASE, Global Health, PsycINFO, the Health Management Information Consortium database [HMIC], Social Policy and Practice, the Cumulative Index to Nursing and Allied Health Literature [CINAHL], the International Bibliography of the Social Sciences [IBSS], Web of Science, Africa Web, Index Medicus for South-East Asia Region [IMSEAR], Index Medicus for the Eastern Mediterranean Region [IMEMR], and Latin American and Caribbean Health Sciences Literature [LILACS]) were searched using controlled vocabulary terms and key/text words from first record to 23 October 2013. Terms for IPV and MSM were adapted from Cochrane protocols and peer-reviewed systematic reviews [24]–[27]. Terms for mental disorders were adapted from a review by Trevillion and colleagues [5]. No language restrictions were applied. Reference lists of all included studies were also searched and backward and forward citation tracking used to identify additional potentially relevant studies. Three LGBT specialised journals that featured in our searches (*Journal of Homosexuality*, *Journal of Gay & Lesbian Social Services*, and *Journal of LGBT Issues in Counseling*) were hand searched. Furthermore, experts were asked to identify additional studies. An example search strategy is provided in Text S1, and the protocol of the review can be found in Text S2.

### Inclusion and Exclusion Criteria

**Figure 1:**
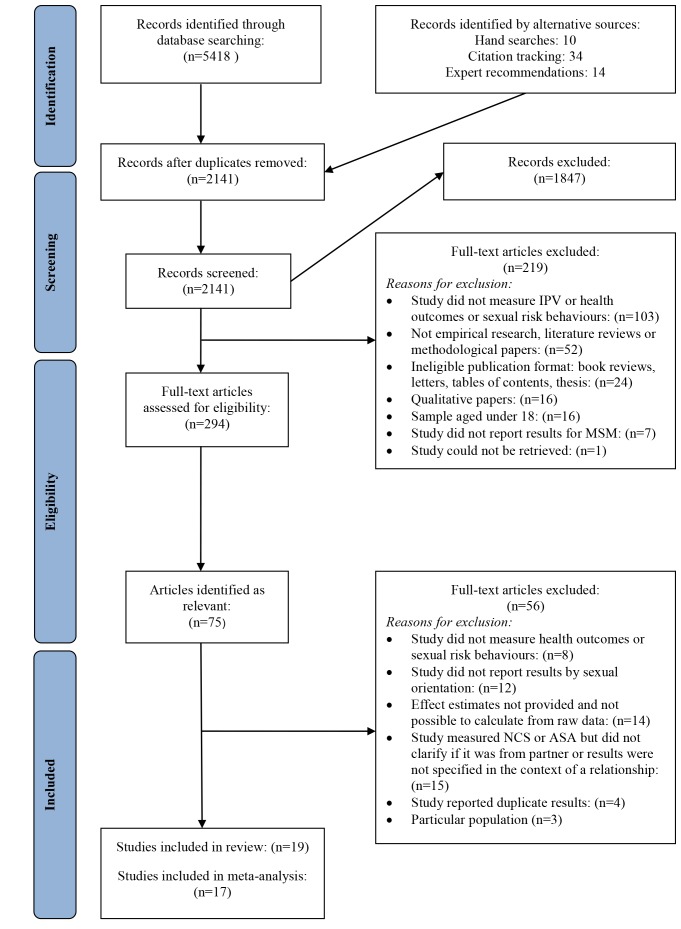
Flow diagram of screened and included papers. ASA, adult sexual assault; NCS, non-consensual sex.

The study selection process is summarised in the flowchart in Figure 1. Studies were eligible for inclusion if they (a) measured IPV in any of its forms (physical, emotional, and/or sexual); (b) reported results for men who defined themselves as gay or bisexual and/or reported having had sex with men, and who were 18 y or older; (c) were cohort studies, case-control studies, or cross-sectional studies; and (d) measured the prevalence and odds or risk of the health outcomes selected for inclusion in this review, or reported data from which these statistics could be calculated. Studies were excluded if they (a) reported on adult sexual assault or non-consensual sex outside of an intimate relationship; (b) reported IPV in a specific group that made it difficult to generalise the results to the wider population, such as IPV in the armed forces, among prison inmates, or in HIV positive individuals. Health conditions included in the review were selected based upon the most frequent outcomes associated with IPV in the broader literature [11],[20],[28]–[30]. They were classified in two groups: health conditions (depression, anxiety, and post-traumatic stress symptoms; suicide ideation and suicide attempt; substance use; eating disorders; STIs; and HIV) and sexual risk behaviours (unprotected receptive or insertive anal intercourse and number of sexual partners).

### Screening and Data Extraction

AM. B. and L. J. B. screened the abstracts and full texts of potentially eligible studies. Details of the 1,848 excluded papers and reasons for exclusion were documented. Data from included papers were extracted by AM. B. and L. J. B. into a customised Excel spreadsheet. Extracted data included the following: study design; sample characteristics; definitions and measures of IPV; the health conditions and sexual risk behaviours measured; and their effect estimates and measures of uncertainty. Details about confounders controlled for were also recorded.

### Quality Appraisal

The quality of each association estimate was appraised by AM. B. and L. J. B. using criteria adapted from validated tools [31] and STROBE (Strengthening the Reporting of Observational Studies in Epidemiology) [32]. An appraisal checklist for included studies was developed. The checklist provided explanatory notes for extracting each data element to ensure consistency between reviewers (see Text S3). The inter-rater agreement rate between reviewers for the quality appraisal was high (*K* = 0.86), and discrepancies were resolved through discussion with a third reviewer (K. M. D.).

### Data Analyses

All analyses were conducted by K. M. D. and AM. B using Stata 12.0 [33]. Descriptive statistics on study characteristics and quality are presented. We computed an overall quality score (Table 1) for descriptive purposes, but in accordance with recommended practice, this was not used to weight studies in the meta-analysis [34]. Random effects meta-analysis was used to calculate the pooled prevalence of different forms of violence and to calculate pooled odds ratios (ORs) representing associations between exposure to IPV and various health outcomes and sexual risk behaviours. Where studies did not report prevalence estimates or ORs, these were calculated from raw data where possible. One study [35] reported a risk ratio, which was converted to an OR using the formula of Zhang and Kai [36]. Higgins's *I* ^2^ statistic, which describes the percentage of variability in point estimates that is due to heterogeneity rather than sampling error [37], was calculated. We also calculated confidence intervals for each *I* ^2^ statistic using the *heterogi* command in Stata [38]. Following other reviews 25%, 50%, and 75% were taken to indicate low, moderate, and high levels of heterogeneity, respectively [39],[40]. Meta-regression analyses were run in order to establish predictors of heterogeneity.

**Table 1:** Quality assessment of estimates in studies included in the review.

Study	Sample	Response Rate	Bias	Missing Data	Study Participants	MSM Definition	IPV Measure	Outcomes Measure	Precision of Effect Measure	Transparency of Results	Adjusted Analysis	Overall Quality Score^a^ (Maximum 22)
Bartholomew et al. (2008) [42]	1	2	2	0	2	0	2	0	2	1	1	13
Dunkle et al. (2013) [44]	0	0	0	0	2	2	1	0	2	2	2	11
Dyer et al. (2012) [46]	0	2	0	0	2	2	0	1	2	2	2	13
Feldman et al. (2007) [35]	0	2	0	0	2	0	2	0	2	1	1	10
Greenwood et al. (2002) [47]	1	2	0	0	2	2	2	0	2	0	1	12
Houston and McKirnan (2007) [54]	0	0	0	2	2	2	1	1	2	1	1	12
Hughes et al. (2010) [48]	2	2	0	0	2	0	0	2	2	2	0	12
Kelly et al. (2011) [49]	0	2	0	0	2	0	2	0	2	1	0	9
Koblin et al. (2006) [50]	0	2	1	0	2	2	1	1	2	2	0	13
Li et al. (2012) [57]	0	2	0	0	2	0	1	2	2	2	1	12
Mustanski et al. (2007) [55]	0	0	0	2	2	0	0	1	2	2	2	12
Mustanski et al. (2011) [45]	0	0	0	0	2	2	2	1	2	2	2	13
Nieves-Rosa et al. (2000) [56]	0	0	0	0	2	2	0	1	2	0	2	9
Stall et al. (2003) [51]	1	2	0	0	2	2	2	2	2	0	2	15
Stephenson et al. (2010) [52]	0	2	0	0	2	2	1	0	2	0	0	9
Stephenson et al. (2011) [43]	0	0	0	0	2	0	2	0	2	2	2	10
Stephenson et al. (2011) [58]	0	2	0	0	2	2	2	0	2	2	2	14
Welles et al. (2011) [60]	0	0	0	0	2	2	2	0	2	2	2	12
Wong et al. (2010) [53]	0	0	0	0	2	2	2	0	2	2	2	12

a A key with the criteria for scoring each quality category is provided in Text S3.

Some studies reported results in multiple publications or had multiple estimates using overlapping measures of either violence or health outcomes. To avoid double-counting participants, which can lead to falsely precise pooled estimates, we selected only the least biased estimate per study. Decisions were based on the quality criteria described above and the following algorithm: preference was given to multivariate estimates over bivariate, to where the reference group was unexposed to any violence, and to where the estimate was most precise (i.e., smallest confidence interval). To investigate small study effects and possible publication bias, we constructed contour funnel plots in Stata [41] (Figure S1). For this we used all available estimates for a given outcome (rather than choosing one per study).

## Results

### Study Characteristics

Nineteen studies describing 18 datasets (Greenwood et al. [47] and Stall et al. [51] are based on the same dataset) with 13,797 participants, and reporting 87 estimates, met the inclusion criteria; 17 studies were included in the meta-analysis. Sixteen studies were conducted in the US, one in Canada [42], one in South Africa [43], and one in China [44] (study characteristics are shown in Tables 2 and S2). The mean age of participants ranged from 19 [45] to 47 [46] y old. Average participation rate was 63%, with 11 studies [35],[42],[46]–[52],[57],[58] reporting it.

**Table 2:** Characteristics of studies included in the review.

Study	Country	Sample (*N* a)	IPV Types and Measures Used	Outcomes Measured
Bartholomew et al. (2008) [42]	Canada	Household probability telephone sample (186 MSM).	Experience and perpetration of physical and psychological IPV measured with a modified version of CTS.	Substance use, HIV status.
Dunkle et al. (2013) [44]	China	Respondent-driven sample (404 Chinese MSM and male sex workers).	Experience of physical, emotional, and sexual IPV measured by specific behavioural items except for sexual IPV, which was measured by a single behaviour item.	Sexual risk behaviours
Dyer et al. (2012) [46]	US	Clinical setting (301 black MSM).	Experience of physical, mental, or emotional IPV measured by single behaviour items.	Depression symptoms, stress, substance use, HIV status, and high-risk sex.
Feldman et al. (2007) [35]	US	Community-based convenience sample (912 Latino gay and bisexual men).	Experience of physical, psychological, and sexual IPV measured by specific behavioural items.	HIV sexual risk behaviour.
Greenwood et al. (2002) [47]	US	Household probability telephone sample (2,881 MSM).	Experience of physical, psychological, and sexual IPV measured with modified version of CTS.	HIV status.
Houston and McKirnan (2007) [54]	US	Community-based convenience sample (817 MSM).	Experience of physical, verbal, and sexual IPV measured by specific behavioural items except for sexual IPV, which was measured by a single behaviour item.	Substance use, depression, HIV status, sexual behaviour.
Hughes et al. (2010) [48]	US	Population-based random sample (338 gay, bisexual, and “not sure” men).	Experience of physical IPV measured by single behaviour item.	Substance use disorder.
Kelly et al. (2011) [49]	US	Community-based convenience sample (1,782 gay and bisexual men).	Experience and perpetration of physical and emotional IPV measured with an adapted version of the Greenwood and colleagues [47] scale, which was a modified version of CTS.	Substance use.
Koblin et al. (2006) [50]	US	Community-based convenience sample (539 MSM).	Experience of physical and emotional IPV measured by specific behavioural items for physical violence and a single behaviour item for emotional violence.	Club drug use, HIV status, UAS.
Li et al. (2012) [57]	US	Clinic-based convenience sample (2,295 MSM).	Experience of physical, verbal, and sexual IPV measured by specific behavioural items except for sexual IPV, which was measured by a single behaviour item.	Substance use, HIV status.
Mustanski et al. (2007) [55]	US	Community-based convenience sample (288 MSM).	Experience of physical and emotional IPV measured by single behavioural items.	Substance use, HIV status, UAS.
Mustanski et al. (2011) [45]	US	Community-based convenience sample (413 MSM).	Experience of physical and sexual IPV measured by specific behavioural items.	Drug use, UAS.
Nieves-Rosa et al. (2000) [56]	US	Community- based convenience sample (273 MSM).	Experience of psychological, physical, and sexual IPV measured by single behaviour items.	Substance use, UAS.
Stall et al. (2003) [51]	US	Household probability telephone sample (2,881 MSM).	Experience of physical, symbolic, and sexual IPV measured with a modified version of the CTS.	Substance use, depressive symptoms, HIV status, UAS.
Stephenson et al. (2010) [52]	US	Online-based convenience sample (665 gay or bisexual men).	Experience and perpetration of physical and sexual IPV; sexual IPV was measured by a single behaviour item.	HIV status.
Stephenson et al. (2011) [43]	South Africa	Online-based convenience sample (521 MSM).	Experience and perpetration of physical or sexual IPV measured by specific behaviour items.	UAS.
Stephenson et al. (2011) [58]	US	Online-based convenience sample (528 MSM).	Experience and perpetration of physical, psychological, and sexual IPV measured with CTS-Revised.	HIV status.
Welles et al. (2011) [60]	US	Clinical-based setting (128 black MSM).	Experience and perpetration of physical and sexual IPV measured by specific behavioural items.	Substance use.
Wong et al. (2010) [53]	US	Community-based sample (526 MSM).	Experience and perpetration of physical, emotional, and sexual IPV measured with an adaptation of the Women's Experience with Battering Scale [59].	Substance use.

a *N* based on participants included in the calculation of the estimates included in the review.

Table 1 summarises estimates' quality. Most studies used convenience, non-probabilistic samples, with the exception of one study that used a population-based sample [48] and three studies that used random samples of particular cities in the US and Canada [42],[47],[51]. Response bias was addressed in two studies [42],[50]. Similarly, two studies [54],[55] reported their missing data policy or performed a sensitivity analysis. All the estimates reported or calculated from the raw data had uncertainty measures, and four studies [47],[51],[52],[56] did not report their non-significant results. Four studies [48]–[50],[52] did not control for confounders within the analyses, or we calculated the estimates from raw data and did not control for confounders. Confounders included a wide range of variables, with most studies controlling for age, ethnicity, level of education, substance use, and HIV status.

Only nine of 19 studies used validated self-report measures of depressive symptoms [46],[51],[54], substance use [48],[51],[54], or sexual risk behaviours [45],[48],[55],[56], or biological samples to establish HIV seropositivity [50],[51],[57]. Measures were otherwise unvalidated self-report measures for health conditions, including HIV status. Studies included in the review reported on different populations: gay only, gay and bisexual, MSM only, MSM and bisexual, and LGBT. Most (12) studies used behavioural measures to define their study populations as MSM. Seven studies [35],[42],[43],[48],[49],[55],[57] relied on self-reported sexual orientation to define their study population.

The recall times for which IPV was measured included “lifetime prevalence”, “past 5 years”, “in the last 12 months”, “in the past three months”, and “in your current relationship”. Five studies [42],[47],[49],[51],[58] reported using an adapted version of the Conflict Tactics Scale (CTS), and one [53] used an adapted version of a scale by Smith and colleagues [59]. Four studies used unique IPV measures developed by the authors that included multiple behaviours to measure all the types of IPV included in the study [35],[43],[45],[60], five studies used multiple behaviour measures for physical IPV but not for emotional or sexual violence [44],[50],[52],[54],[57], and four studies used single behaviour items to measure all types of IPV included in the study [46],[48],[55],[56]. Physical violence was measured in all 19 studies (13,812 participants). Emotional violence included verbal and psychological violence, and it was measured in 14 studies [35],[42],[44],[46],[47],[49]–[51],[53]–[58] (11,732 participants), and sexual violence was measured in 13 studies [35],[43],[44],[45],[47],[51]–[54],[56]–[58],[60] (10,363 participants).

### Intimate Partner Violence Victimisation: Prevalence and Health Associations

Combined measures of any lifetime violence (physical, sexual, or emotional) were reported in six studies [35],[50],[53]–[56] with 3,355 participants. Estimates ranged from 32% [54] to 82% [53], with a pooled prevalence of 48% (95% CI_p_ 31.23–64.99) and a high level of heterogeneity between pooled estimates (*I* ^2^ = 95.7, 95% CI*_I_* ^2^ 92%–98%). Removal of the outlying estimate [53] did not improve heterogeneity. When asking about experience of any kind of IPV in the last 5 y, a pooled prevalence of 31.95% (95% CI_p_ 19.32–44.58) in four studies [44], with 7,362 participants was found. Estimates ranged from 16% to 51%, and there was a high level of heterogeneity between estimates (*I* ^2^ = 99.3, 95% C*_I_* ^2^ 99%–100%) (Table 3). Eight studies included prevalence measures of any type of IPV victimisation [58],[60], physical violence only [48], or a combination of two types of violence [43],[45],[46],[50],[52], with various recall periods.

**Table 3:** Pooled prevalence for IPV victimisation.

Recall Period	Any Violence				Physical Violence				Sexual Violence				Emotional Violence			
	Studies (*n*)	*N*	Prevalence (95% CI)	Heterogeneity (95% CI)	Studies (*n*)	*N*	Prevalence (95% CI)	Heterogeneity (95% CI)	Studies (*n*)	*N*	Prevalence (95% CI)	Heterogeneity (95% CI)	Studies (*n*)	*N*	Prevalence (95% CI)	Heterogeneity (95% CI)
**Lifetime**	5**^a^**	2,829	41.24	95.7%	6	3,405	24.05	95.5%	4	2,528	14.60	91.1%	5	3,067	34.44	97.1%
			(32.38–50.11)	(92%–98%)			(17.41–30.69)	(92%–97%)			(9.88–19.32)	(80%–96%)			(24.61–44.28)	(95%–98%)
**5 y**	4	7,362	31.95	99.3%	2	5,176	19.5	95.2%	3	5,580	5.1	0%	2	5,176	25.50	99.5%
			(19.32–44.58)	(99%–100%)			(14.60–24.40)	(NA)^b^			(4.52–5.67)	(0%–90%)			(8.84–42.15)	(NA)
**Past year**	0	0	NA	NA	3	1,729	13.99	96.1%	3	1,729	7.31	80.9%	1	665	31.99	NA
			(NA)	(NA)			(6.08–21.89)	(92%–98%)			(4.53–10.09)	(40%–94%)			(24.42–39.55)	(NA)

a Without outlier.

b It was not possible to calculate the 95% CI because of the small number of studies.

NA, not available.

**Figure 2:**
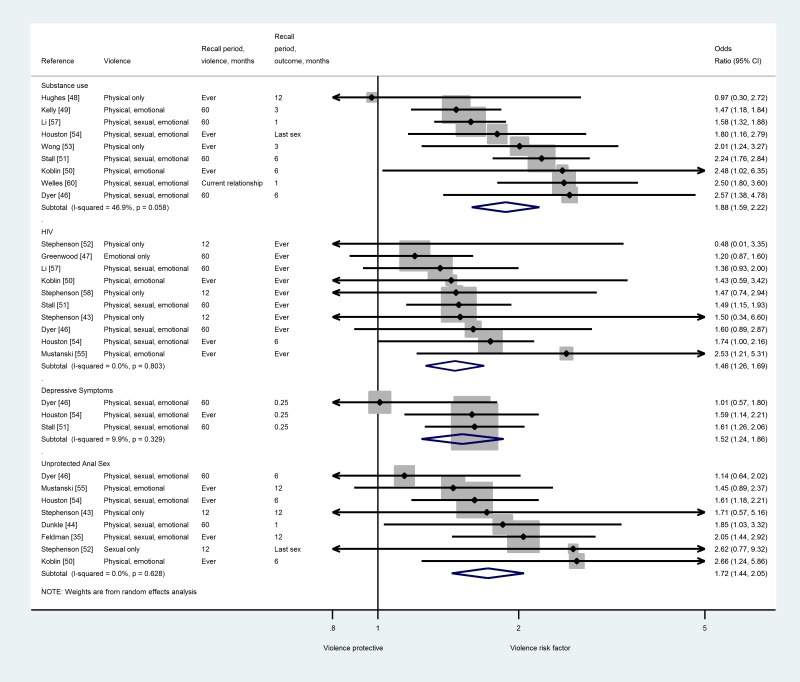
Meta-analysis of the association between IPV and health outcomes and sexual risk behaviours in MSM who are victims of IPV.

Estimates of the association between IPV victimisation and substance use were provided in nine studies [46],[48]–[51],[53],[54],[57],[60] that included 9,607 MSM who had been exposed to any kind of IPV and had consumed different kinds of substances (Figure 2). We found that exposure to IPV was associated with increased odds of substance use, with an OR for pooled estimates of 1.88, (95% CI_OR_ 1.59–2.22), but with moderate heterogeneity between studies (*I* ^2^ = 46.9%, 95% CI*_I_* ^2^ 0%–78%) (Table 4). In order to investigate sources of heterogeneity for this meta-analysis we ran meta-regressions to test the impact of IPV modality and type of drug consumed on heterogeneity. Our results showed that none of these variables explained the heterogeneity (Table 5).

**Table 4:** Random-effects pooled odds ratios for health associations (victimisation and perpetration).

Health Outcome	Victimisation: Any Violence				Perpetration: Any Violence			
	Studies (*n*)	*N*	OR (95% CI)	Heterogeneity (95% CI)	Studies (*n*)	*N*	OR (95% CI)	Heterogeneity (95% CI)
**Health Condition**								
Substance use	9	9,607	1.88	46.9%	2	1,910	1.99	73.1%
			(1.59–2.22)	(0%–78%)			(1.33–2.99)	(NA)
HIV	10	8,835	1.46	0.00%	3	1,729	0.93	0.00%
			(1.26–1.69)	(0%–62%)			(0.49–1.78)	(0% to 90%)
Depression	3	3,999	1.52	9.9%	0	—	—	—
			(1.24–1.86)	(0%–91%)				
**Sexual Risk Behaviour**								
UAS^a^	8^a^	4,447	1.72	0.00%	2	1,064	1.88	80.2%
			(1.44–2.05)	(0%–68%)			(0.22–16.03)	(NA)

a Without outlier.

NA, not available.

**Table 5:** Meta-regression exploring potential sources of heterogeneity in substance use meta-analysis.

Characteristic	OR (95% CI)	*p*-Value
Physical or emotional violence versus any violence	0.79 (0.48–1.31)	0.308
Alcohol use versus any other substance use	1.44 (0.81–2.52)	0.174
Drug use versus any other substance use	1.30 (0.85–1.97)	0.183

Estimates for the odds of HIV were provided in ten studies [43],[46],[47],[50]–[52],[54],[55],[57],[58] that included 8,835 MSM who had been exposed to any kind of IPV (Figure 2). Exposure to IPV was associated with a positive HIV status, with a pooled OR of 1.46 (95% CI_OR_ 1.26–1.69) and high consistency between pooled estimates (*I* ^2^ = 0.0%, 95% CI*_I_* ^2^ 0%–62%) (Table 4).

MSM exposed to any kind of violence also had increased odds of reporting depression symptoms. Depression symptoms were measured in three studies including 3,999 MSM using the Center for Epidemiologic Studies Depression Scale (CESD) [46],[51],[54], with a pooled OR of 1.52 (95% CI_OR_ 1.24–1.86, *I* ^2^ = 9.9%, 95% CI*_I_* ^2^ 0%–91%) (Figure 2; Table 4). Estimates for unprotected anal sex (UAS) were calculated in nine studies [35],[44]–[46],[50],[52],[54],[56],[58]. After running the meta-analysis and removing an outlier study [56], we kept eight estimates with a total population of 4,447 MSM (Figure 2). We found that exposure to IPV was associated with increased odds of engaging in UAS (pooled OR = 1.72, 95% CI_OR_ 1.44–2.05, *I* ^2^ = 0.0%, 95% CI*_I_* ^2^ 0%–68%) (Table 4). Contour-enhanced funnel plots showed no evidence of publication bias for substance use, HIV, and UAS results (Figure S1). In these plots studies are missing from the left side of the graph, but across areas of both statistical significance and non-significance. This suggests that publication bias is not responsible for funnel plot asymmetry [41]. The small number of studies that could be pooled for the depression symptoms meta-analysis precluded the use of such plots [46],[61],[62] for this health correlate.

### Intimate Partner Violence Perpetration: Prevalence and Health Associations

Prevalence rates of IPV perpetration among MSM were reported in seven studies [42],[43],[49],[52],[53],[58],[60] with a total population of 4,336 MSM. These studies reported prevalence for different types of violence and different recall periods, precluding the possibility of meta-analysis. Estimates for the odds of substance use among perpetrators were provided in two studies [49],[60] that included 1,910 MSM who disclosed perpetration of IPV. Perpetration of IPV was associated with increased odds of substance use (pooled OR = 1.99, 95% CI_OR_ 1.33–2.99) (Table 4). The results showed moderate heterogeneity (*I* ^2^ = 73.1%). Due to the small number of studies, CI*_I_* ^2^ could not be calculated for this result. Likewise, it was not possible to assess publication bias. No other significant associations between IPV perpetration and health outcomes or sexual risk behaviours were found when conducting the meta-analyses (Table 4).

## Discussion

### Main Findings

Our findings suggest that IPV victimisation is prevalent among MSM. We also found evidence that exposure to IPV as a victim is associated with increased odds of substance use, depressive symptoms, being HIV positive, and UAS among MSM. Relatively few studies measured perpetration of IPV, but these studies suggested an association between IPV perpetration and substance use. However, these results should be interpreted with caution given the methodological limitations in the included studies.

The associations of IPV with both UAS and being HIV positive suggested in this review are consistent with the existence of a causal pathway. Emotional, physical, or sexual violence (or a combination of all three) could increase risk for UAS [28],[63], which in turn could increase risk for HIV infection. Further research is needed to investigate this link, including the specific associations between different types of IPV and HIV status. In particular, the intersection of IPV and HIV seropositivity might be problematic. MSM may fear partner violence following disclosure of HIV status and may experience difficulties adhering to treatment regimes and accessing health services. Sexual health and HIV services are opportune points of intervention for MSM affected by IPV [64], and these findings underscore the need for these services to provide comprehensive sexual health care and awareness of repeat STI or positive HIV status as indicators for IPV and vice versa among the MSM population. Our findings on the association of substance use and depression symptoms with IPV confirm previous findings [48],[65] and also highlight the need for mental health and substance misuse services to address IPV issues. Men who are depressed or engage in heavy substance use and are also part of a violent relationship might find it hard to seek help, to end the relationship, or adhere to prescribed treatment.

### Strengths and Limitations of This Review

To our knowledge this is the first systematic review to quantitatively summarise the associations between IPV victimisation and perpetration with various health conditions in MSM. Our review entailed extensive searches of the international research literature without any time or language restrictions, and most of our results were consistent and showed no publication bias. Despite this, the review has some limitations. First, prevalence results are only for studies included in the review and should be interpreted with caution. Second, we did not contact authors for additional data. Third, for studies that did not provide ORs, it was not possible to control for confounders when ORs were calculated from raw data [48]–[50],[52].

### Limitations of Included Studies

The included studies had a number of methodological weaknesses, and the search produced few published studies addressing the association between IPV and health among MSM. Similar to other meta-analyses with a low number of studies, confidence intervals for the heterogeneity estimates were very wide [66]. This means that if further studies were to be conducted on the associations between IPV and health outcomes among MSM the results could be more homogenous or more heterogeneous. Heterogeneity in our review could have been due to different factors such as different recall periods, types of IPV included, the use of convenience samples, different definitions of sexual identity, and the quality of tools used to assess IPV and health outcomes. Unfortunately, tests for sources of heterogeneity have low power with small numbers of studies.

Furthermore, all studies included were cross-sectional, and there was a lack of uniformity regarding the recall periods for IPV and key outcomes, which made it impossible to assess the temporality of the associations. Most studies used convenience, non-probabilistic samples, which makes it difficult to generalise the results to the wider population. Few studies used validated tools to measure IPV, health outcomes, or sexual risk behaviours. HIV measurement relied mainly on self-report, which can provide a biased measure of HIV prevalence. There was a lack of consistency in the types of IPV measured in the studies; however, most authors used either a modified version of CTS or specific behavioural acts to measure IPV. Studies used scales that have been validated for heterosexual samples, which did not necessarily capture MSM's experience of IPV, and most studies used an author's IPV measure without discussing issues of validity or psychometric characteristics. Measures of sexual violence were limited and usually included a single behaviour item asking about forced or unwanted sex, thus potentially resulting in an underestimation of this type of abuse. Additionally, most of the IPV was measured without considering contextual factors such as the frequency with which the abuse occurred or its perceived impact [67]. A step towards a validated tool for IPV among MSM in the US has been taken by Stephenson and Finneran [19], which may be useful in future studies.

Another limitation arises from the fact that we included studies that used different kinds of sexual identities (i.e., self-recognised as gay or bisexual, reporting same-sex sexual encounters, or both). Men's experiences of IPV and the impact it has on their health may be affected by their identification as gay or not, which in turn might be related to their levels of internalised homophobia. Further studies including a differentiated analysis by sexual identity and sexual behaviour might help to understand these relationships better. One such study found that men who identified themselves as gay had a higher prevalence of IPV compared to men who disclosed same-sex sexual behaviour, but did not identify themselves as gay or bisexual [18]. The term “partner” was not clearly operationalised in many of the studies. Studies measuring IPV often define “partner” as someone with whom the respondent has had sex. Therefore, some studies may have captured violence and abuse experienced in the context of casual encounters. This may have diluted the association between health outcomes and IPV because it is less likely that these relationships are characterised by a pattern of abuse and coercive, controlling behaviour that escalates over time [68],[69]. Moreover, the role of sexual agreements, which can be monogamous or non-monogamous [70] and are common among gay couples [70],[71], was missing from the studies identified. Lastly, there was a dearth of research relating to MSM in low- and middle-income countries [72], where MSM are at elevated risk of HIV infection and in some countries might face serious discrimination and multiple barriers to accessing health care [73].

### Implications for Research

Future research should include national studies using probability-based samples to establish more reliable estimates of the prevalence of different types of IPV experience and perpetration amongst MSM. Further studies should try to establish the impact of particular (more common) forms of IPV among MSM in order to clarify the connections between specific forms of violence and health conditions. Additionally, the sexual orientation and identity of participants should be clearly established, and analysis should differentiate between the two. This will help determine whether the factors that drive IPV and the consequences of IPV are different across men who identify as gay or bisexual compared with men who have same-sex sexual behaviours but do not identify themselves as gay or bisexual.

Longitudinal studies are needed to establish the causal pathway between IPV and adverse health outcomes and sexual risk behaviours. The concepts of bi-directional violence or reciprocity of violence, common in the literature on IPV in MSM, should be taken into consideration in future research. Future studies should also use measurement tools validated for MSM populations and prospective biological measures of STIs and HIV. Other health outcomes and health risk behaviours for which we did not find any studies, such as eating disorders, repeated STIs, sexual compulsivity [74], and concurrent sexual partnerships [70],[71],[75], may also be important in understanding the impact that IPV has for this particular population. Furthermore, in view of the increasing evidence that the chronic stress of both childhood and adulthood abuse is associated with inflammatory markers that increase the risk of long term diseases including cardiovascular disease and diabetes [76],[77], longer term adverse health outcomes are also possible and should be measured in future studies.

### Implications for Practice

The findings of this review underscore the need for health professionals to be aware that IPV is a problem for the MSM population and to carefully assess for IPV and refer affected individuals to appropriate support services in the community. Therefore, same-sex IPV awareness training for health professionals caring for this population is needed. This training should include developing skills for asking about IPV [78],[79], dealing with partner notification and safety issues, and making referrals to specialist IPV support services [80]. However, the availability of local and national support services for MSM experiencing or perpetrating IPV varies considerably internationally, and there are very limited data available on their effectiveness [81]. From a health services perspective, adequate protocols for dealing with IPV should be in place, ensuring that all primary and secondary health care services provide holistic care. Addressing IPV is a global health priority, and rigorous evaluations of the feasibility and effectiveness of health service interventions for IPV amongst MSM are needed to inform future policy and practice.

## Supporting Information

Figure S1 **Funnel plots to assess publication bias.**
(DOCX)

Table S1 **PRISMA checklist.**
(DOC)

Table S2 **Characteristics of studies included in the review.**
(DOCX)

Text S1 **Example search strategy.**
(DOCX)

Text S2 **Systematic review protocol.**
(DOCX)

Text S3 **Critical appraisal checklist for included studies.**
(DOCX)

## Abbreviations

CTS: Conflict Tactics Scale
IPV: intimate partner violence
LGBT: lesbian, gay, bisexual, and transgender
MSM: men who have sex with men
OR: odds ratio
STI: sexually transmitted infection
UAS: unprotected anal sex
