# Supplementary material for: Associations between Intimate Partner Violence and Health among Men Who Have Sex with Men: A Systematic Review and Meta-Analysis
Source: PLoS Med. 2014 Mar 4;11(3):e1001609. doi: 10.1371/journal.pmed.1001609 (PMC3942318; doi:10.1371/journal.pmed.1001609)
Supplement: Figure S1 — Funnel plots to assess publication bias. (DOCX) [file pmed.1001609.s001.docx]

**Figure S1: Funnel plots to assess publication bias.**

Figure 1 Bias assessment plot for odds of substance use

Figure 2: Bias assessment plot for of odds of HIV positive status

Figure 3 Bias assessment plot for odds of unprotected anal sex
