# Supplementary material for: Associations between Intimate Partner Violence and Health among Men Who Have Sex with Men: A Systematic Review and Meta-Analysis
Source: PLoS Med. 2014 Mar 4;11(3):e1001609. doi: 10.1371/journal.pmed.1001609 (PMC3942318; doi:10.1371/journal.pmed.1001609)
Supplement: Table S2 — Characteristics of studies included in the review. (DOCX) [file pmed.1001609.s003.docx]

Table S2: Characteristics of studies included in the review

| Author | Country  and cities | Sample | Participants  Characteristics | IPV measures used | Outcomes definition and measures used |
| --- | --- | --- | --- | --- | --- |
| Bartholomew et al (2008)^42^ | Canada, Vancouver. | Household probability telephone sample. | 186 MSM.  Mean age 38.53 (SD=9.44).  46%British/English/Scottish/Welsh/Irish.  27% Other European.  12% Other Canadian.  15% Other nationalities.  42% Some or all of university education.  30% Some or all of a community college/technical school education.  14% Some or all of postgraduate education. | CTS. Men asked if they had been recipient of 13 acts of psychological abuse and 14 acts of physical abuse from a male partner in the last 12 months.  Men asked if they had perpetrated the same acts against a male partner in the last 12 months. | **Substance use:** Drinking level assessed as the product of frequency (on a nine point scale from never to once per day) and quantity per occasion.  Self-report on the use of 11 illegal recreational drugs over the past year.  **HIV status:** Self-reported. |
| Dunkle et al (2013)^44^ | China, Shangai | Respondent driven sample. | 404 Chinese MSM and male sex workers.  Mean age 29.6 (10.4)  36.8% Middle school  40% High School  23.1% College | Participants were asked about the number of different boyfriends or partners who had ever perpetrated a range of violent behaviours including: being hit, being the target of thrown objects, being threatened with harm or harm others, being threatened with having their sexuality revealed, being physically forced to have sex, or destruction of property. | **Sexual risk behaviours:** assessed using 16 items. |
| Dyer et al (2012)^46^ | USA, Baltimore MD, Chicago IL, Los Angeles CA, Pittsburgh PA. | Clinic-based convenience sample. | 301 Black MSM  Mean age 47 (SD=9.0)  17.3% High School or less  26.6% Some college  24.9% College graduate  31.2% Postgraduate | Defined as any reported experience of physical, mental or emotional abuse over the past 5 years perpetrated by a boyfriend or other male sexual partner. | **Depression symptoms:** CES-D score.  **Stress:** 14 item scale asked participants to indicate level of stress over the past 12 months.  **Substance use**: Self-reported use of one or more of the following: at least weekly use of poppers, crack,methamphetamine, cocaine, heroin, speedball, and/or ecstasy in the past 6 months.  **HIV status:** Lab tests.  **High risk sex:** self-report of unprotected anal intercourse (insertive and receptive) in the past 6 months. |
| Feldman et al (2007)^35^ | USA, Los Angeles, Miami and New York. | Community-based probability sample of Latin and gay venues. | 912 Latino gay and bisexual men.  Mean age 31.2 (SD not provided).  They were all of Latino origin with 72% not born in the U.S. | **Psychological IPV:** ‘Have you ever been insulted or verbally abused by a lover or boyfriend?’  **Physical IPV:** ‘Have you ever been hit, kicked, or slapped by a lover or boyfriend/girlfriend?’  **Sexual IPV:** ‘Have you ever been sexually abused or raped by a lover or boyfriend/girlfriend?’ | **HIV sexual behaviour:** Self-report of unprotected anal intercourse with a casual sex partner or with a lover boyfriend with whom they did not have a monogamous arrangement.  Participants were asked about their two more recent sexual partners within the last 12 months. |
| Greenwood et al (2002)^47^ | U.S., four urban centres. | Household probability telephone sample. | 2881 MSM.  79% White.  10% Latino  4% African American  4% Asian/Pacific Islander  3% Native American  45% College degree  30% High school diploma or less  25% Graduate/professional degree | Modified version of the conflict tactic scale (CTS) to be culturally specific to MSM.  They also developed two ‘global’ measures of battering victimisation. | **HIV status:** Self-reported. |
| Houston & McKirnan (2007)^54^ | U.S., Chicago | Community-based convenience sample at 11 diverse gay/bisexual venues. | 817 men.  Mean age 33 (SD=9.8).  51% African-American.  22% White.  16% Latinos.  10% Asian/Pacific Islanders and other  Median education: some college. | **Physical IPV:** ‘Have you ever had unwanted physical harm within a relationship?’ (I.e. been hit, kicked, shoved, burned, cut, or facing other undesired physical harm).  **Verbal IPV:** ‘Have you ever experienced unwanted physical and sexual threats, physical humiliation, or control in a relationship?’  **Sexual IPV:** ‘Have you ever felt forced to have unwanted sexual contact in a relationship?’  Participants indicated whether each form of abuse had occurred in a current relationship, a past relationship, or both. | **Substance use:** General use of each of 11 substances (e.g. alcohol, marijuana, cocaine) was assessed via seven-point frequency ratings ranging from ‘never’ to ‘about daily’.  **Depression:** Centre for Epidemiological Studies’ Depression Scale (CES-D).  **HIV status:** Self-reported**.**  **Sexual behaviour:** Self-report on how many men they had sex with during the past 6 months. |
| Hughes et al (2010)^48^ | U.S. | Population based random sample. | 338 gay, bisexual, not sure men  39% 25-44 years old  35% 45-62 years old  19% > 65 years old  8% 20-24 years old  71% White  12% Hispanic  11% Black  4% Asian/Pacific Islander  2% Native American  59% More than high school  28% Completed high school  14% Less than high school | IPV was assessed by asking:  ‘Were you ever physically attacked or badly beaten up by your spouse o romantic partner?’ | **Substance use disorder (SUD):** Alcohol Use Disorders and Associated Disabilities Interview Schedule DSM-IV (AUDADIS-IV) symptom questions were used to operationalise DSM-IV abuse and dependence for 10 substances in the past 12 months.. |
| Kelly et al (2011)^49^ | U.S., New York City, Los Angeles. | Community-based convenience sample from GLB events. | 1782 gay and bisexual men.  Mean age 37.07 (range 18-78, SD not provided).  57% White.  20% Latino.  12% Asian/Pacific Islanders and other.  11% Black.  37% Bachelor’s degree.  28% Some college/Associate’s degree  24% Graduate degree.  11% Up to H.S. diploma. | IPV was measured with an adapted version of Greenwood and colleagues^34^ which used an adapted version of the CTS themselves.  **Physical IPV:**  ‘Hit you with fists or open hand?’  ‘Thrown something at you?’  **Non-Physical:**  ‘Verbally threatened you in any way?’  ‘Damaged or destroyed your property?’  Participants asked if they had experienced any of these in the past 5 years by a primary partner.  Similar questions asked regarding perpetration of violence.  Issues of mutual partner violence were explored. | **Substance use:** Participants were asked if they had used a substance or more from a list of alcohol and several illicit drugs.  They also were asked if they had been treated for substance use. |
| Koblin et al (2012)^50^ | U.S., New York city | Community-based convenience sample from public venues frequented by young MSM. | 539 MSM men.  40.4% 19-22 years old.  41% Latino.  24% African-American.  17% Mixed race/ethnicity.  12% White.  6% Asian/Pacific Islanders and men from the Caribbean.  No education information. | **Threats of physical IPV:**  ‘Have you ever been threatened or intimidated by a lover or partner?’  **Physical IPV:**  ‘Have you ever been hit/kicked/punched or otherwise physically hurt by an exchange partner, non-steady partner or steady partner? Is it happening now?’ | **Club drug use:** Use of any of the following drugs in the last 6 months: ecstasy, amphetamines, barbiturates, hallucinogens, poppers or cocaine.  **HIV:** Antibody test results.  **Unprotected anal sex:** Self-report of having any anal sex without a condom in the last six months. |
| Li Ying et al (2012)^57^ | U.S., Washington DC. | Clinic-based convenience sample. | 2295 MSM men.  Median age 30 (range 15-78, SD not provided).  65% Caucasian.  16% African-American.  10% Hispanic.  9% All other.  No education information. | IPV was evaluated through a questionnaire with 11 items specific for the client’s relationship experience within the last 5 years.  **Physical IPV:**  ‘Have you ever been hit with a fist or open hand?’ ‘Pushed or shoved?’ ‘Forced to get high or drunk?’ ‘Kicked?’ ‘Stalked?’ ‘Having something thrown at you?’ ‘Having your property damaged or destroyed?’’  **Verbal IPV** (threats of physical/sexual violence + psychological/emotional violence):  ‘Have you ever been verbally threatened in any way?’ ‘Verbally demeaned in front of any stranger?’ ‘Made fun of the appearance?’  **Sexual IPV:**  ‘Have you ever been forced to have sex?’ | **Substance use:** Recreational drug use was measured as use of poppers, stimulants, both or none of them in the past 30 days.  **HIV:** Use of HIV enzyme-linked immunosorbent assay (ELISA) antibody testing. With confirmatory Western blot in Clinical Laboratory Improvement Amendments (CLIA). |
| Mutanski et al (2007)^55^ | U.S., Chicago | Community-based convenience sample. | 288 MSM men.  Median age 20 (SD=2.4) and 54% were under 21.  33% Black.  30% White.  26% Hispano/Latino.  8% Other/Multiracial.  3% Asian/Pacific Islander.  No education information. | IPV assessed with three items asking about experiences of being threatened, physically hurt, or bullied by a same-sex romantic partner. | **Substance use:** Assessed with the AIDS-Risk Behaviour Assessment (ARBA).  **HIV status:** Self-report. Have you ever been told by a doctor that you have HIV?  **Unprotected anal sex:** self-reported inconsistent condom use for receptive or insertive anal sex in the last 12 months. |
| Mutanski et al (2011)^45^ | U.S., Large Midwestern city. | Community-based convenience sample. | 413 MSM men.  Mean age 18.53 (range 16-20, SD=1.21). 23% were under age 18.  49% African American.  19% White.  12% Latino/Hispanic.  12% Multiracial.  6% Other.  No education information. | **Physical IPV:**  **‘**Has your partner ever hit, slapped, punched or hurt you?’  **Forced sex:**  **‘**Did this partner ever force you to have vaginal, anal, or oral sex when you didn’t want to?’    In this context ‘force’ was defined as physical and nonphysical pressure, such as pushing you, arguing with you or threatening you in order to have sex. | **Drug use:** assessed with the AIDS-Risk Behaviour Assessment (ARBA).  **Unprotected anal sex:** number of unprotected anal or vaginal sex acts within each sexual partnership. |
| Nieves-Rosa et al (2000)^56^ | U.S.,  New York metropolitan area. | Community- based convenience sample | 273 MSM who had had a committed relationship.  Mean age 31 (range 18-56, SD not provided).  Sample from men born in Colombia, Dominican Republic, Mexico or Puerto Rico, anybody born in the U.S. and self-identified as Latino or Hispanic with a least one parent or both grandparents born in any of the included countries.  No education information. | **Domestic abuse:** Defined as psychological, physical, or sexual abuse perpetrated by one partner with the intention of intimidating or harming him.  **Physical abuse:**  ‘Have you ever been harmed by physical aggression inflicted on you by any of your partners?’  **Psychological abuse:**  ‘Have you ever been intimidated or harmed by any of your partners?’  **Sexual abuse:**  ‘Have you ever been forced by any of your partners to have receptive anal sex without condoms after 1981?’  Participants were also asked if they considered they had been victims of partner abuse in any of their relationships | **Substance use:** Alcohol and drug use was assessed by providing participants with a list that included up to fifteen recreational drugs.  **Unprotected sex:** Measured using the Sexual Practices Assessment Schedule (SPAS). Inventory, explores frequency of unprotected sex over the previous 12 months. |
| Stall et al (2003)^51^ | U.S., Chicago, Los Angeles, New York and San Francisco | Household probability telephone sample. | 2881 MSM men.  16% older than 50 years old.  21% Black.  No education information. | Modified version of the conflict tactic scale. IPV was measured as the experience of any form of violence: Symbolic, physical or sexual in the past 5 years with a primary partner | **Substance use:** Polydrug use measured as the use of 3 or more recreational in the last 6 months.  **Depressive symptoms:** Measured by CES-D scale. Scores >22 considered as depression.  **HIV serostatus:** Not specified how they measured it.  **Unprotected anal sex**: Unprotected sex with a partner of known discordant or unknown status. (last four partners). |
| Stephenson et al 2010^52^ | U.S. | Online-based convenience sample using MySpace.com. | 665 gay or bisexual men.  68% 18-24 years old.  21% 25-29 years old.  11% 30-35 years old.  48% White/Caucasian.  38% Hispanic.  15% Black/African American.  69% Some college or higher.  27% High School/General Educational Development.  5% Less than high School/General Educational Development. | **Physical IPV:**  ‘In the last 12 months has any partner been physically violent to you? This includes pushing, holding you down, hitting you with his fist, kicking, attempting to strangle, attacking with a knife, gun or other weapon.’  **Sexual IPV:**  ‘In the last 12 months has any partner ever forced you to have sex when you were unwilling?’  Men were also asked if they had perpetrated physical or sexual violence towards a male partner. | **HIV status:** Patients were asked about their HIV testing history.  **Sexual risk behaviours**: Participants were asked if last anal sex was unprotected. |
| Stephenson et al (2011a)^43^ | South Africa. | Online-based convenience sample using Facebook.com. | 521 MSM.  40% 25-34 years old.  29% 16-24 years old.  20% 35-44 years old.  11% 45+ years old.  90% White/European/African.  5% Black African.  3% Coloured.  1% Asian.  1% Other.  58% < 12 years.  42% > 12 years. | Participants were asked if they had experienced or perpetrated physical IPV or sexual IPV in the 12 months prior to the survey.  **Physical IPV:**  ‘In the last 12 months has any partner been physically violent to you? This includes pushing, holding you down, hitting you with his fist, kicking, attempting to strangle, attacking with a knife, gun or other weapon.’  **Sexual IPV:**  ‘In the last 12 months has any partner ever forced you to have sex when you were unwilling?’ or ‘used threats to make me have oral or anal sex.’  Men were also asked if they had perpetrated any form of IPV against their current male partner. | **Unprotected anal sex:** measured as recent unprotected anal sex. |
| Stephenson et al (2011b)^58^ | U.S. | Online-based convenience sample using Facebook.com. | 528 MSM men.  84% White.  82% Had at least some college education. | Conflict Tactics Scale Revised used to assess both experience and perpetration of IPV. |  |
| Welles et al (2011)^60^ | U.S., Boston. | Clinic-based convenience setting. | 128 black MSM men.  30% 36-45 years old.  26% 26-35 years old.  23% 46+ years old.  23% 18-25 years old.  No education information. | IPV experience and perpetration were assessed specific to physical or sexual violence in current relationships.  **Physical IPV perpetration:**  ‘Have you ever hit, slapped, punched, shoved, choked, kicked, shaken, or otherwise physically hurt your current partner?’  **IPV injury perpetration:**  ‘Has your partner ever had any injuries, such as bruises, cuts, black eyes, or broken bones as a result of being hurt by you?’  **Sexual IPV perpetration:**  ‘Have you made your partner take part in any sexual activity that she did not want to, including touching that made him or her feel uncomfortable?’  ‘Have you forced or pressured your partner to have vaginal, oral or anal sex with you?’  Being a victim of IPV was assessed by the same four related questions in the recipient position. | **Substance use:** For each type of substance, subjects were asked if they had used the following in the past 30 days: alcohol (to intoxication), marijuana, cocaine, and/or heroin. |
| Wong et al (2010)^53^ | U.S., Los Angeles county. | Community-based convenience sample in gay identified venues. | 526 MSM men.  Average age 20.1 (SD not provided)  39% were 18-19 years old.  39% Latino of Mexican descent.  37% Caucasian.  24% African American. | Adaptation of Smith, Earp and DeVellis scale to IPV among battered women. Scale included 12 items that assess whether participants have ever been victims of physical, emotional and sexual abuse. In addition 3 items assessed if they had ever perpetrated intimate partner physical violence (alpha = 0.83). | **Substance use:** Assessed as recent (past 3 months) use of illicit drugs such as crack, LSD, PCP, mushrooms, cocaine, crystal/methamphetamine, other stimulants, ecstasy, GHB, ketamine, poppers, and prescription drugs used without a physician’s order. |
