# Supplementary material for: Associations between Intimate Partner Violence and Health among Men Who Have Sex with Men: A Systematic Review and Meta-Analysis
Source: PLoS Med. 2014 Mar 4;11(3):e1001609. doi: 10.1371/journal.pmed.1001609 (PMC3942318; doi:10.1371/journal.pmed.1001609)
Supplement: Text S1 — Example search strategy. (DOCX) [file pmed.1001609.s004.docx]

**Text S1: Example search strategy**

**Medline (OVID) (1946 to October 2013)**

1 Domestic Violence/

2 Spouse Abuse/

3 ((violence or abus*) adj3 (domestic or partner or spous* or dating or relationship or physical or sexual* or

verbal* or emotional* or psychological*)).mp.

4 1 or 2 or 3

5 Homosexuality/

6 Homosexuality, Male/

7 Transsexualism/

8 Bisexuality/

9 Homosexual*.mp.

10 Transexual*.mp.

11 Bisexual*.mp.

12 Transgender.mp.

13 (MSM or men who have sex with men or ((man or men or male*) adj3 (gay or homosexual or queer or

bisexual* or transsexual* or transgender)) or LGBT).mp.

14 5 or 6 or 7 or 8 or 9 or 10 or 11 or 12 or 13

15 exp Sexually Transmitted Diseases/

16 exp HIV/

17 exp HIV-2/

18 exp HIV-1/

19 exp HIV Infections/

20 sexually transmitted infection*.mp.

22 mental disorders/

23 exp anxiety disorders/

24 anxiety/

25 depressive disorder/

26 depression/

27 exp stress disorders, traumatic/

28 stress, psychological/

29 exp "sexual and gender disorders"/

30 mental disorder*.mp.

31 anxiety.mp.

32 anxiety disorder*.mp.

33 depressive disorder*.mp

34 depressi*.mp.

35 stress disorder*.mp.

36 post traumatic stress disorder*.mp.

37 suicide/

38 suicidal ideation/

39 suicide, attempted/

40 suicid* ideation.mp. [mp=title, abstract, original title, name of substance word, subject heading word,

protocol supplementary concept, rare disease supplementary concept, unique identifier]

41 suicide attempt*.mp. [mp=title, abstract, original title, name of substance word, subject heading word,

protocol supplementary concept, rare disease supplementary concept, unique identifier]

42 suicid* behavio$r*.mp. [mp=title, abstract, original title, name of substance word, subject heading word,

protocol supplementary concept, rare disease supplementary concept, unique identifier] (3394)

43 exp substance-related disorders/

44 drinking behavio$r/

45 alcohol drinking/

46 drug-seeking behavio$r/

47 Substance-related disorder*.mp.

48 Drug-seeking behavio$r*.mp.

49 ((abuse or misuse or behavior or drink*) adj3 (alcohol or substance or drug*)).mp.

50 exp eating disorders/

51 eating disorder*.mp.

52 anorexia.mp.

53 anorexia nervosa.mp.

54 bulimia.mp.

55 15 or 16 or 17 or 18 or 19 or 20 or 21 or 22 or 23 or 24 or 25 or 26 or 27 or 28 or 29 or 30 or 31 or 32 or

33 or 34 or 35 or 36 or 37 or 38 or 39 or 40 or 41 or 42 or 43 or 44 or 45 or 46 or 47 or 48 or 49 or 50 or

51 or 52 or 53 or 54

56 4 and 14 and 55

57 exp Risk-Taking/

58 Sexual Behavio$r/

59 exp Sexual Partners/

60 Unsafe sex/

61 Sexual partners.mp.

62 Unsafe sex.mp.

63 (risk taking adj3 sexual behavio$r).mp.

64 unprotected sex.mp.

65 unprotected anal intercourse.mp.

66 (risk taking adj3 condom).mp.

67 57 or 58 or 59 or 60 or 61 or 62 or 63 or 64 or 65 or 66

68 4 and 14 and 67
