# Supplementary material for: Associations between Intimate Partner Violence and Health among Men Who Have Sex with Men: A Systematic Review and Meta-Analysis
Source: PLoS Med. 2014 Mar 4;11(3):e1001609. doi: 10.1371/journal.pmed.1001609 (PMC3942318; doi:10.1371/journal.pmed.1001609)
Supplement: Text S2 — Systematic review protocol. (DOCX) [file pmed.1001609.s005.docx]

**Text S2: Systematic Review Protocol**

1. **Title**

Associations between Intimate Partner Violence and Health among Men Who Have Sex with Men: A Systematic Review and Meta-Analysis

1. **Review Team**

Dr. Ana Maria Buller

Dr. Loraine J Bacchus

Dr. Karen M Devries.

Professor Louise M Howard.

1. **Background**

Historically, intimate partner violence (IPV) research has focused on women as recipients of violence and abuse from male partners. Population based surveys from around the world demonstrate that IPV against women is extremely prevalent affecting 1 in 3 women in high income countries, with higher rates reported in low and middle income countries (1-3). The associated adverse health consequences for women victims are well documented and include depression, anxiety, post traumatic stress disorder, eating disorders (4-5), and sexual and reproductive health problems (6-7).

However, IPV is not exclusive to opposite-sex relationships and there is a growing body of research highlighting the prevalence of domestic/partner violence in same sex relationships [(8-13)](#_ENREF_8). Recent reviews suggest that the prevalence in same-sex couples, in particular bisexual men and men who have sex with men (MSM), is as high as or higher than it is for women in opposite-sex relationships [(14-16)](#_ENREF_14). The reported lifetime experience of IPV among MSM lies between 15.4% and 51% (10, 12, 14). This high variation is a result of the methodological challenges of studying the MSM population, such as the lack of population based samples (12, 16) and varying definitions of ‘partner’ or ‘relationship’[(12)](#_ENREF_12). Most reviews addressing the LGBT (lesbian, gay, bisexual and transgender) population focus on the prevalence of IPV violence, with limited research of the effects of IPV on same-sex couples’ health [(17)](#_ENREF_24). This systematic review and meta-analysis therefore aims to quantitatively synthesise the existing evidence on the association between exposure to IPV and health outcomes and sexual risk behaviours among MSM.

1. **Objectives**

The objectives of this review are to establish if:

1. MSM who experience or perpetrate IPV have increased odds of STI’s, HIV, eating disorders, common mental disorders or substance misuse compared with MSM who are not experiencing or perpetrating IPV.
2. MSM men who experience or perpetrate IPV engage more in sexual risk-taking behaviours compared to those who are not experiencing or perpetrating IPV.

The key terms of the review are defined as follows:

**Men who have sex with men**

Men who self-identify as gay or bisexual or men who do not identify necessarily as gay or bisexual but report having had or having other men as sexual partners.

**Intimate Partner Violence**

Intimate partner violence (IPV) refers to any “incident or patterns of incidents of controlling, coercive or threatening behavior, violence or abuse (psychological, physical, sexual, financial or emotional) between those aged 16 or over who are, or have been intimate partners or family members regardless of gender or sexuality”[(18)](#_ENREF_1). For the purposes of this review the term IPV will refer or focus on violence and abuse that occurs in the context of an intimate relationship among those aged 18 or over.

**Health outcomes**

Health outcomes, for the purpose of this review, are defined as including the following conditions identified from the literature:

*Depressive symptoms*

*Anxiety symptoms*

*Suicide ideation report*

*Post traumatic stress symptoms*

*Eating disorder symptoms*

*Substance use*

*STI (Sexually transmitted disease)*

*HIV status*

**Sexual risk behaviours**

*Unprotected anal receptive and insertive sex*

*Multiple partners*

*Sero-discordant sexual relationships*

1. **Selection Criteria**

**5.1 Inclusion criteria**

Study Population

Studies will be eligible for inclusion in the review if samples include men who self-identify as gay or bi-sexual or report having sex with men, who are 18 years or older and who are experiencing or have experienced IPV as victims and/or perpetrators.

Study Setting

No restrictions have been placed on study setting.

Study Characteristics

Studies will be eligible for inclusion if they present the results of peer-reviewed research based on the following study designs: cohort studies, case-control studies, or cross-sectional studies. Non-English literature will be included.

Study Outcomes

Studies will be eligible for inclusion in the review, if they measure the odds or risk of the selected health outcomes or sexual risk behaviours, or report data from which these statistics can be calculated.

**5.2 Exclusion criteria**

Study Population

Studies will be excluded if they: (a) include participants who are aged 17 years or younger and do not provide appropriate age-disaggregated data; (b) include participants who are women or men in opposite sex relationships and do not provide appropriate sexual orientation-disaggregated data; (c) report on adult sexual assault or non consensual sex outside of an intimate relationship; (d) report IPV in a specific group which makes it difficult to generalise the results to the wider population such as IPV in the armed forces or among prison inmates.

Study Characteristics

Studies using any of the following research designs will be excluded: case studies, case series, or any qualitative design (e.g. ethnography, in-depth interviews, focus groups). General discussion papers, comments, letters, book chapters, theses/dissertations, and conference papers will be excluded.

Study Outcomes

Studies which do not measure the risk (i.e. odds ratios, relative risk) of health outcomes and sexual risk behaviours (as defined in section 4) amongst MSM who experience or perpetrate IPV (as defined in section 4) will be excluded from the review.

1. **Search Strategy**

The review will attempt to locate all peer-reviewed published studies which meet the above inclusion criteria.

**6.1 Search Terms**

The search terms for MSM were adapted from published Cochrane protocols and peer-reviewed reviews (19-20). Search terms for intimate partner violence are adapted from published Cochrane protocols and peer-reviewed reviews (21-22). Search terms for mental disorders are adapted from published reviews (4) (see Text S1).

**6.2 Data Sources**

The review will search multiple electronic databases up to October 2013 with no lower date limit. Reference lists of all included studies will also be searched and backward and forward citation tracking used to identify additional potentially relevant studies. Hand searching of key journals will be carried out. Key experts in the field will be asked for references.

Electronic Databases

The following databases will be searched:

- MEDLINE
- EMBASE
- Global Health
- Psycinfo
- HMIC
- Social Policy and Practice
- CINAHL
- IBSS (Social Sciences)
- Web of Science
- LILACS (South America)
- IMSEAR (South East Asia)
- IMEMR (Eastern Mediterranean)
- Africa Wide

 No lower date limits or language restrictions will be placed on electronic searches.

Hand Searching

Hand searches of the following journals will be conducted:

- The Journal of Homosexuality
- Journal of Gay & Lesbian Social Services
- Journal of LGBT Issues in Counseling

No lower date limits will be placed on hand searches.

**6.3 Bias**

Publication/Language and Location Bias

Our decision to include studies written in languages other than English will help to address publication bias. As part of the analysis, the reviewers will assess to what extent the review is affected by publication bias.

Duplication Bias

Where the review identifies multiple eligible papers from the same study, only the main paper with the total N containing data of relevance to the objectives of the review will be extracted and included in the analysis.

1. **Conducting the Review**

**7.1 Title and Abstract Screening**

Literature searches of the databases listed in section 6 will be conducted and the resulting citations will be downloaded to EndNote© software, where duplicate citations will be removed. Additional citations that have been identified by forward and backward citation tracking, hand searches and key experts will also be added to the EndNote© database.

Inclusion and exclusion of papers will be based on the criteria described in section 5 titles and abstracts of all downloaded citations. When it is not possible to determine if a citation is relevant, it will be included at this stage. Full hard copies of the papers identified as potentially relevant will be obtained.

**7.2 Retrieval and Screening of Full Text Articles**

Two researchers (AMB and LJB) will then independently assess the selected full papers for eligibility according to the study-eligibility criteria detailed above. Any disagreements at any of the screening stages will be resolved by discussion between the two reviewers in the first instance. If agreement cannot be reached, then a third reviewer (KMD) will independently review full paper, and a majority decision will be taken on inclusion/exclusion.

**7.3 Data Extraction**

Data extraction will be carried out by AMB and LJB using a pro-forma to be developed by AMB for this purpose. Data relating to study authors, study design, year of study, sample characteristics, number of participants, definitions and measures of IPV; health conditions and sexual risk behaviours; and their effects estimates and measures of uncertainty, as well as confounding variables included in the analysis (such as age, sex, ethnicity, SES); will be recorded.

**7.4 Study Appraisal**

Estimates of association will be methodologically appraised by AMB and LJB using criteria adapted from previously validated tools [28], STROBE (Strengthening the Reporting of Observational Studies in Epidemiology) [29], and different checklists [28-29]. A tailored appraisal checklist for included studies will be developed (see Text S3). Reviewers will assess the quality of the estimates independently and inter-rater agreement rate between reviewers will be calculated. Discrepancies will be resolved through discussion between the two reviewers and ultimately with a third reviewer (KMD).

The quality of reporting on the following criteria will be assessed: sampling strategy, response rate, non-response bias, missing data and missing data policy described, study subjects, definition of MSM provided, IPV measure, health conditions or sexual risk behaviour measure, precision of effect measures (confidence intervals and p values given) and use of confounding variables in the analysis.

**7.5 Data Analysis**

The extracted data will be analysed in STATA. Basic descriptive analysis will be conducted to summarise information about the study population (e.g., the country studied, the age range studied, and the type of population covered); the sample characteristics (e.g. the sample size and the response rate); and the method of measuring IPV, the health outcomes and risk behaviours. The risk of health associations and risk behaviours will be reported using the most commonly used measure of effect in the included studies, which we anticipate will be odds ratios. 95% confidence intervals will be reported for all measures of prevalence and odds ratios. The results of the analyses will be tabulated and/or displayed graphically using forest plots. We will use contour funnel funnel plots in order to detect publication bias. Higgins I² statistic will assist the analysis of the heterogeneity of the included studies, with values of p<0.10 taken as indicative of heterogeneity. Confidence intervals will be computed for each I² statistic.

Random-effect meta-analysis will be considered if there are sufficient studies available with suitable data. Meta-regression may also be considered if there are sufficient studies with similar variables available that might enable combined analysis of factors associated with combined outcomes.

**References**

1. Devries K, Mak J, García-Moreno C, Petzold M, Child J, Falder G, et al. The Global Prevalence of Intimate Partner Violence Against Women. Science. 2013.

2. Garcia-Moreno C, Jansen HAFM, Ellsberg M, Heise L, Watts CH. Prevalence of intimate partner violence: findings from the WHO multi-country study on women's health and domestic violence. The Lancet. 2006;368(9543):1260-9.

3. Walters M, Chen J, Breiding M. The National Intimate Partner and Sexual Violence Survey (NISVS): 2010 Findings on Victimization by Sexual Orientation. Atlanta, GA: National Center for Injury Prevention and Control, Centers for Disease Control and Prevention2013.

4. Trevillion K, Oram S, Feder G, Howards L. Experiences of domestic violence and mental disorders: a systematic review and meta-analysis. PLOS one2012.

5. Ellsberg M, Jansen HAFM, Heise L, Watts CH, Garcia-Moreno C. Intimate partner violence and women's physical and mental health in the WHO multi-country study on women's health and domestic violence: an observational study. The Lancet. 2008;371(9619):1165-72.

6. Sharps PW, Laughon K, Giangrande SK. Intimate Partner Violence and the Childbearing Year: Maternal and Infant Health Consequences. Trauma, Violence, & Abuse. 2007 April 1, 2007;8(2):105-16.

7. Coker AL. Does physical intimate partner violence affect sexual health? A systematic review. Trauma, Violence, & Abuse. 2007;8(2):149-77.

8. Alexander CJ. Violence in Gay and Lesbian Relationships. Journal of Gay & Lesbian Social Services. 2002 2002/07/10;14(1):95-8.

9. Burke LK, Follingstad DR. Violence in lesbian and gay relationships: Theory, prevalence, and correlational factors. Clinical Psychology Review. 1999 August;19(5):487-512.

10. Henderson L. Prevalence of domestic violence among lesbians and gay men. London: Sigma Research. 2003.

11. Mak WW, Chong ES, Kwong MM. Prevalence of same-sex intimate partner violence in Hong Kong. Public Health (Elsevier). 2010;124(3):149-52.

12. Nowinski SN, Bowen E. Partner violence against heterosexual and gay men: Prevalence and correlates. Aggression and Violent Behavior. 2012 January;17(1):36-52.

13. Owen SS, Burke TW. An exploration of prevalence of domestic violence in same-sex relationships. Psychological Reports. 2004 August;95(1):129-32.

14. Stanley JL, Bartholomew K, Taylor T, Oram D, Landolt M. Intimate violence in male same-sex relationships. Journal of Family Violence. 2006;21(1):31-42.

15. Merrill GS, Wolfe VA. Battered gay men: an exploration of abuse, help seeking, and why they stay. Journal of Homosexuality. [Research Support, Non-U.S. Gov't]. 2000;39(2):1-30.

16. Finneran C, Stephenson R. Intimate Partner Violence Among Men Who Have Sex With Men A Systematic Review. Trauma, Violence, & Abuse. 2013;14(2):168-85.

17. Randle AA, Graham CA. A Review of the Evidence on the Effects of Intimate Partner Violence on Men. Psychology of Men & Masculinity. 2011;12(2):97-111.

18. Office H. Domestic Violence and Abuse. 2013; Available from: https://www.gov.uk/domestic-violence-and-abuse.

19. Johnson Wayne D, Diaz Rafael M, Flanders William D, Goodman M, Hill Andrew N, Holtgrave D, et al. Behavioral interventions to reduce risk for sexual transmission of HIV among men who have sex with men. Cochrane Database of Systematic Reviews [serial on the Internet]. 2008; (3): Available from: http://onlinelibrary.wiley.com/doi/10.1002/14651858.CD001230.pub2/abstract.

20. Zhang L, Qian H-z, Blevins Meridith L, Yin L, Ruan Y, Vermund Sten H. Internet-based behavioral interventions for preventing HIV infection in men who have sex with men (MSM). Cochrane Database of Systematic Reviews [serial on the Internet]. 2011; (12): Available from: http://onlinelibrary.wiley.com/doi/10.1002/14651858.CD009525/abstract.

21. Coulthard P, Yong Sin L, Adamson L, Warburton A, Worthington Helen V, Esposito M, et al. Domestic violence screening and intervention programmes for adults with dental or facial injury. Cochrane Database of Systematic Reviews [serial on the Internet]. 2010; (12): Available from: http://onlinelibrary.wiley.com/doi/10.1002/14651858.CD004486.pub3/abstract.

22. Ramsay J, Carter Y, Davidson L, Dunne D, Eldridge S, Hegarty K, et al. Advocacy interventions to reduce or eliminate violence and promote the physical and psychosocial well-being of women who experience intimate partner abuse. Cochrane Database of Systematic Reviews [serial on the Internet]. 2009; (3): Available from: http://onlinelibrary.wiley.com/doi/10.1002/14651858.CD005043.pub2/abstract.
