# Supplementary material for: Associations between Intimate Partner Violence and Health among Men Who Have Sex with Men: A Systematic Review and Meta-Analysis
Source: PLoS Med. 2014 Mar 4;11(3):e1001609. doi: 10.1371/journal.pmed.1001609 (PMC3942318; doi:10.1371/journal.pmed.1001609)
Supplement: Text S3 — Critical appraisal checklist for included studies. (DOCX) [file pmed.1001609.s006.docx]

**Text S2: Critical appraisal checklist for included studies (0 to 2 points per criteria. 22 points maximum score)**

1. **SAMPLE:** Are subjects representative of the population?

Purposive, clinical and community settings, public venues, self-selected = 0

Representative at the local level = 1

Population based = 2

1. **RESPONSE RATE**: Is the response rate reported?

No= 0

Yes/Not applicable (all participants answered) = 2

1. **NON-RESPONSE BIAS:** Is response bias measured? Is there any response bias?

If not reported/not measured = 0

If response bias measured = 1

+

Are non respondents different from respondents? If they are the same = + 1 (2 in total)

If they are different = + 0 (1 in total)

1. **MISSING DATA:** Is the missing data reported and missing data policy described?

If not mention to missing data = 0

If missing data excluded = 0

If sensitivity analysis showed that respondents with missing data and non missing data were different = 1

If there is no missing data = 2 (i.e. interviewer/computer made sure all the questions were answered)

If data imputed= 2

If sensitivity analysis showed that respondents with missing data and non missing data are the same = 2

1. **STUDY SUBJECTS:** Are the study subjects described in detail? (At least age, ethnicity and SES or education level described?)

No = 0

Yes = 2

1. **DEFINITION OF MSM PROVIDED:** Is MSM status measured by same sex sexual behaviour? Or does the setting guarantee same-sex sexual activity (i.e. gay sexual health clinic)?

No (only self-identification as gay or bisexual measured) = 0

Yes = 2

1. **IPV MEASURE:** Is IPV measured by specific acts, or a validated tool?

None of the IPV was measured by specific acts or a validated tool = 0

Some of them = 1

Yes, all of them = 2

1. **ACCURATE HEALTH CONDITIONS OR SEXUAL RISK BEHAVIOURS MEASURES:** Are the health conditions or sexual risk behaviour measures accurate (valid and reliable), for instance use of a validated tool (mental health, substance use), or biological measure (STI, HIV)?

None of the health outcomes was measured accurately (researchers own questions, self-report only) = 0

Some of them = 1

Yes, all of them = 2

**9.** **PRECISION OF EFFECT MEASURES:** Are the significant effect estimates provided with uncertainty measures (i.e. p value or confidence intervals)?

None of the estimates include uncertainty measures = 0

Some of the estimates include uncertainty measures = 1

Yes, all of the estimates include uncertainty measures = 2

1. **TRANSPARENCY OF RESULTS:** Are statistically non-significant results presented?

None non-significant results presented = 0

Non-significant results presented but without uncertainty measures = 1

Only uncertainty measures presented without the values for the non-significant results = 1

Yes, non-significant results presented with uncertainty measures = 2

1. **ADJUSTED ANALYSIS:** Were any confounding variables controlled for in the analyses?

No = 0

All estimates were calculated for this review, so no confounding variables considered = 0

Yes, only one confounding variable considered or some confounding variable considered for some estimates and no confounding variables considered for other estimates = 1

Yes, more than one confounding variable considered for all estimates = 2
